# Bithionol inhibits ovarian cancer cell growth *In Vitro* - studies on mechanism(s) of action

**DOI:** 10.1186/1471-2407-14-61

**Published:** 2014-02-04

**Authors:** Vijayalakshmi N Ayyagari, Laurent Brard

**Affiliations:** 1 Division of Gynecologic Oncology; Department of Obstetrics and Gynecology, Southern Illinois University School of Medicine, Springfield, IL, USA

**Keywords:** Bithionol, Ovarian cancer cell lines, Apoptosis, Reactive oxygen species, Autotaxin

## Abstract

**Background:**

Drug resistance is a cause of ovarian cancer recurrence and low overall survival rates. There is a need for more effective treatment approaches because the development of new drug is expensive and time consuming. Alternatively, the concept of ‘drug repurposing’ is promising. We focused on Bithionol (BT), a clinically approved anti-parasitic drug as an anti-ovarian cancer drug. BT has previously been shown to inhibit solid tumor growth in several preclinical cancer models. A better understanding of the anti-tumor effects and mechanism(s) of action of BT in ovarian cancer cells is essential for further exploring its therapeutic potential against ovarian cancer.

**Methods:**

The cytotoxic effects of BT against a panel of ovarian cancer cell lines were determined by Presto Blue cell viability assay. Markers of apoptosis such as caspases 3/7, cPARP induction, nuclear condensation and mitochondrial transmembrane depolarization were assessed using microscopic, FACS and immunoblotting methods. Mechanism(s) of action of BT such as cell cycle arrest, reactive oxygen species (ROS) generation, autotaxin (ATX) inhibition and effects on MAPK and NF-kB signalling were determined by FACS analysis, immunoblotting and colorimetric methods.

**Results:**

BT caused dose dependent cytotoxicity against all ovarian cancer cell lines tested with IC_50_ values ranging from 19 μM – 60 μM. Cisplatin-resistant variants of A2780 and IGROV-1 have shown almost similar IC_50_ values compared to their sensitive counterparts. Apoptotic cell death was shown by expression of caspases 3/7, cPARP, loss of mitochondrial potential, nuclear condensation, and up-regulation of p38 and reduced expression of pAkt, pNF-κB, pIκBα, XIAP, bcl-2 and bcl-xl. BT treatment resulted in cell cycle arrest at G1/M phase and increased ROS generation. Treatment with ascorbic acid resulted in partial restoration of cell viability. In addition, dose and time dependent inhibition of ATX was observed.

**Conclusions:**

BT exhibits cytotoxic effects on various ovarian cancer cell lines regardless of their sensitivities to cisplatin. Cell death appears to be via caspases mediated apoptosis. The mechanisms of action appear to be partly via cell cycle arrest, ROS generation and inhibition of ATX. The present study provides preclinical data suggesting a potential therapeutic role for BT against recurrent ovarian cancer.

## Background

Ovarian cancer accounts for 5% of cancer deaths among women in the United States and has the highest mortality rate of all gynecologic cancers [1]. The majority of women diagnosed with advanced ovarian cancer have a low overall survival [2]. Drug resistance is the key reason for ovarian cancer recurrence and poor overall survival [2-4]. Although most ovarian cancer patients (70–80%) initially respond to cytoreductive surgery and adjuvant paclitaxel and platinum-based chemotherapy, the majority will experience disease recurrence [5,6]. The response rate to current second-line or third-line (after interim non-platinum therapy) chemotherapy is less than 33% due to the rise of resistance to these drugs [7-10]. Hence there is a need for more effective therapies and/or treatment approaches to overcome drug resistance.

New drug discovery demands enormous cost and time. An alternative approach is ‘Drug Repurposing’ wherein clinically approved drugs for one indication are re-explored for new applications. It is well known that many drugs exhibit polypharmacological properties, and hence can be explored for their ability to modulate new/alternate targets. ‘Drug repurposing’ is a cost effective alternative to new drug discovery as ADME and basic toxicity are already well established and can be immediately taken to Phase II/III clinical trials. However, in order to “repurpose” these drugs for novel targets/diseases, it is essential to first understand the basic biological action(s) and mechanism(s) of action in preclinical and animal models.

In our present study, we focused on Bithionol (2, 2′-Sulfanediylbis (4, 6-dichlorophenol), a clinically approved anti-parasitic drug as an anti-ovarian cancer drug. Bithionol (BT) has received Food and Drug Administration approval as a second-line orally administered medication for the treatment of helminthic infection and has been safely dosed in humans [11]. All the details of toxicology and pharmacokinetic properties for BT are available (Toxnet, National library of medicine).

BT was shown to be an effective anti-cancer agent in preclinical models and is safe in non-cancer patients [11-13]. BT was shown to decrease tumor weight in a breast cancer model and reduced metastases of tumors initiated with A2058 melanoma cells [12]. BT was reported to reduce melanoma cell migration in a dose-dependent fashion when assayed using *in vitro* cell migration and invasion systems [13]. Similar observations were reported in the case of breast and ovarian cancer cell lines [13]. BT was also reported to show an inhibitory effect on cervical cancer cell growth during *in vitro* screening [14]. These previous studies have proposed possible mechanisms of action of BT against cancer cells. Autotaxin (ATX) inhibition was proposed as a mechanism of action to decrease tumor in a pre-clinical melanoma model [12,13]. An additional mechanism was inhibition of NF-kB signalling via inhibition of IκBα phosphorylation and caspase 3/7 induction [14]. Based on these significant observations, we seek a better understanding of the effect BT on ovarian cancer cell lines, and specifically on cisplatin-resistant cell lines.

The objective of the present study was to explore the cytotoxic effects of BT against ovarian cancer cell lines and to further delineate the cellular mechanism(s) of cytotoxicity. First, we studied the cytotoxic effect (IC_50_ determination) against a panel of ovarian cancer cell lines exhibiting varying sensitivities to cisplatin. Secondly, we identified the type of cell death induced by BT i.e. apoptosis vs. necrosis, by assessment of caspase 3/7 activity and cleaved PARP expression (indicators of apoptosis) and lactate dehydrogenase activity (necrosis marker). In addition to these markers of cell death, we looked at other apoptosis-specific nuclear changes such as chromatin condensation as well as changes in mitochondrial potential.

To further delineate the mechanism(s) of action of BT, we focused on cell cycle, ROS generation, ATX inhibition, and pro-survival (pAkt, pNF-κB p65) and pro-apoptotic signalling (pP38 MAPK) markers. To assess whether BT-induced growth inhibition of the cells is mediated via alterations in cell cycle regulation, we evaluated the effect of BT on cell cycle distribution. Because the production of lethal levels of ROS has been suggested as a mechanism of action of various cytotoxic agents in cancer cells, we assessed effect of BT on ROS generation in ovarian cancer cell lines. To define the cellular response of ovarian cancer cell lines to treatment with BT, we analysed the expression and/or activation of cellular markers that are hallmarks of pro-survival (pAkt, pNF-κB p65) and pro-apoptotic signalling (pP38 MAPK) in all cell lines. Finally, we studied the effect of BT on ATX secretion in ovarian cancer cell lines because BT has been shown to inhibit solid tumor growth in several preclinical cancer models by targeting autotaxin [12,13].

## Methods

### Cell lines and chemicals

In order to assess the cytotoxic effects of BT, a panel of ovarian cancer cell lines exhibiting varying degrees of sensitivities to cisplatin was selected. OVACAR-3 and SKOV-3 are cisplatin-resistant whereas A2780 and IGROV-1 represent cisplatin-sensitive cell lines. Additionally, cisplatin-resistant variants of A2780 and IGROV-1 derived by *in vitro* selection with cisplatin were also tested for BT cytotoxicity. A2780, A2780-CDDP and IGROV-1, IGROV-1CDDP represents isogenic ovarian cancer cell line pairs consisting of a cisplatin-sensitive parental line and a stable cisplatin-resistant sub-line derived by *in vitro* selection with cisplatin.

Human ovarian carcinoma cell lines, OVACAR-3, SKOV-3 were obtained from Dr. McAsey (SIU School of Medicine, Springfield, IL). Isogenic ovarian cancer cell lines pairs, e.g., A2780/A2780-CDDP and IGROV-1/, IGROV-1CDDP were received as a generous gift from Dr. Brodsky (Brown University, Providence, RI). All cell lines were maintained in DMEM media (Sigma) supplemented with 10% heat inactivated FBS (Hyclone), 100 IU penicillin (Mediatech) and 100 μg/mL streptomycin (Mediatech). All cell lines were cultured at 37°C in a humidified atmosphere at 5% CO_2_. The cisplatin resistant variants A2780-CDDP and IGROV-1CDDP cells were treated with 3 μM cisplatin every 3rd passage to maintain cisplatin resistance.

Bithionol (2, 2′-Sulfanediylbis (4, 6-dichlorophenol), Rhodamine-123 and propidium iodide were purchased from Sigma (St Louis, MO). Kinase inhibitors such as LY294002, SB203580 were purchased from Promega. All antibodies were purchased from Cell Signaling Technologies, (Danvers, MA). PrestoBlue™ Cell Viability Reagent and ROS Dye - carboxy-H2DCFDA were purchased from Invitrogen (Carlsbad, CA).

### Cell viability assay

Cell viability after BT treatment was determined by PrestoBlue cell viability reagent (Invitrogen) following the manufacturer's instructions. A 20 mM stock of BT was prepared in DMSO and all the working dilutions were prepared in DMEM media. Ovarian cancer cell lines (5 × 10^3^ cells/well) were plated into 96-well flat bottom plates (Corning, Inc., Corning, NY) and incubated for overnight. Cells were treated with different concentrations of BT ranging from 0.178 μM to 400 μM and further incubated for 48 hrs or 72 hrs. At least 4–6 hrs before the end of treatment time, presto blue reagent was added and incubated for total of 48 or 72 hrs and fluorescence measured (540 nm excitation/590 nm emissions). DMSO concentration was corrected to 1% in all wells. Vehicle treated control cells (media with 1% DMSO) were considered as 100% viable against which treated cells were compared. Experiments were performed in triplicate. Data was expressed as mean ± SD of triplicate experiments. Dose response curves to calculate IC_50_ values were plotted using Graph Pad Prism Software.

In order to ascertain role of ROS in BT induced cytotoxicity, we performed cell viability assays in the presence of an antioxidant, ascorbic acid. Cells were pretreated with 1 mM ascorbic acid for 2 hrs before addition of drug and further incubated for 48 hrs with both BT (50 μM or 100 μM) and ascorbic acid (1 mM). Restoration of cell viability was analyzed.

An additional cell viability assay was performed in order to assess role of p38 activation in BT induced cytotoxicity, in presence of the p38 inhibitor SB203580. Cells were treated with BT (100 μM) in presence of 10 μM SB203580 (non-toxic concentration) for 48 hrs and cell viability was determined.

Lastly, to test if Akt inactivation is essential for drug sensitivity in ovarian cell lines treated with BT, a third cell viability assay was performed in order to see if additional pAkt inactivation would further enhance the effectiveness of BT. To look at this, we treated cells with BT in presence or absence of the pAkt inhibitor LY294002 (10 μM).

### Lactate dehydrogenase (LDH) assay (necrosis assessment)

LDH release was measured using CytoTox-One Homogenous Membrane Integrity kit (Promega) following the manufacturer’s instructions. Briefly, 10 × 10^3^ cells/100 μL were plated per well of the 96-well plate and treated with different concentrations of BT ranging from 12.5 μM to 400 μM for 6, 24 and 48 hrs. Following treatment, 100 μL of CytoTox-One reagent was added to each well. After incubation for 10 min at room temperature, the fluorescence intensity (560 nm excitation/590 nm emission) was measured using a fluorescence microplate reader, Fluoroskan (Thermo Scientifics). A maximum LDH release control set (100% LDH release) was generated as reference to calculate the actual %LDH release from each sample. Percent of LDH released from vehicle treated (1% DMSO media) control set is considered as 100% intact or 0% LDH release. All samples were compared against vehicle control. Experiments were performed in triplicate. Data was expressed as mean ± SD of triplicate experiments.

### Caspase 3/7 assay (apoptosis assessment)

Caspase 3/7 activity was measured using Caspase-Glo 3/7 assay kit from Promega, following the manufacturer’s instructions. Briefly, 10 × 10^3^ cells were plated per well of the 96-well plate and treated as described in the LDH assay (see above). Following treatment, Caspase-Glo 3/7 reagent was added and incubated for 30 min. at room temperature. The luminescence intensity was measured using luminometer (luminoskan, Thermo Scientifics). Cells treated with vehicle (1% DMSO media) were considered as control against which treated cells were compared. Experiments were performed in triplicate. Data was expressed as mean ± SD of triplicate experiments. In addition to homogenous caspase 3/7 assessment, we also analyzed expression of effector caspases, e.g., caspase-3 and caspase-7 via immunoblotting using specific antibodies against caspase 3 and 7 (see Western Blot Analysis below).

### Morphological studies to detect apoptosis

To detect nuclear condensation indicative of apoptosis, NucBlue Live Cell Stain (Hoechst 33342) was used (Invitrogen, Carlsbad, CA). Hoechst 33342 is a cell-permeant nuclear counter-stain that emits blue fluorescence when bound to DNA [15]. It is excited by UV light and emits blue fluorescence at 460 nm when bound to DNA. To detect apoptotic specific nuclear changes, cells (1 × 10^5^ cells) were seeded into 12-well plate and treated with sub-cytotoxic BT at concentrations of 25 μM, 50 μM or 100 μM for 6 or 24 hrs. Following treatment, cells were washed with PBS twice and fresh media containing Hoechst (2 drops/mL of media) was added. Cells were incubated 15 min. at 25°C and observed under fluorescent microscope. Representative images were taken with an inverted microscope (Olympus H4-100, CCD camera) and 20× objective. After morphological assessment by nuclear staining, extent of apoptosis was quantified using the TUNEL assay (described below).

### TUNEL assay

DNA fragmentation was detected using the TiterTACS® 2 TdT *In Situ* Colorimetric Apoptosis Detection Kit (Trevigen, Gaithersburg, MD) following the manufacturer’s instructions. Briefly, cells were seeded at a density of 3 × 10^4^ cells/well, into 96-well flat bottom plates and incubated for overnight. Cells were treated with BT as described previously. Following treatment, cells were washed and fixed followed by addition of labeled nucleotides and subsequent detection with HRP – HRP substrate (TACS-Sapphire) system. The absorbance was measured at 450 nm using a microplate reader, Multiskan (Thermo Scientifics).

### Mitochondrial transmembrane depolarization potential assay

Mitochondrial transmembrane depolarization potential was determined by flow cytometry using Rhodamine-123. Ovarian cancer cells (1 × 10^6^) were seeded in a 100 mm^2^ culture dishes and treated with 50 μM or 100 μM BT for 6 or 24 hrs. Following treatment, cells were harvested by trypsinization, washed with PBS (1×), and resuspended in fresh DMEM medium (1 × 10^6^ cells/mL) containing rhodamine 123 at a concentration of 0.5 mg/mL, and incubated for 30 min. at 37°C. The cells were washed twice with DPBS, re-suspended in DPBS and analyzed by flow cytometry (488 nm excitation/520 nm emission). Data was acquired on a BD Accuri C6 flow cytometer (BD Immunocytometry -Systems, San Jose, CA) and analyzed. Twenty thousand cells were analyzed for each sample. Appropriate gating was used to select standardized cell population.

### Cell-cycle analysis

Cell cycle analysis was carried out by flow cytometry. Cells were seeded into 100 mm^2^ tissue culture dishes (1 × 10^6^ cells/dish) and treated with 50 μM BT for 24 hrs. At the end of the incubation period, detached cells were collected in 15 mL polypropylene centrifuge tubes along with the medium; culture dishes were washed once with PBS. Adherent cells were scraped off and combined in the same tube. After centrifugation (250 g, 5 min.), cells were fixed by adding ice-cold 70% ethanol gradually. Following fixation, cells were stained with propidium iodide (50 μg/mL) in presence of 100 μg/mL of RNase for 30 min at 37°C in the dark. Data was acquired on a BD Accuri C6 flow cytometer (BD Immunocytometry Systems, San Jose, CA) and analyzed. Twenty thousand events were analyzed for each sample. Appropriate gating was used to select standardized cell population.

### Estimation of reactive oxygen species (ROS) production

Hydrogen peroxide, hydroxyl radicals and peroxy radicals were detected via carboxy-H2DCFDA using flow cytometry. Cells (1 × 10^6^) were seeded in a 100 mm^2^ culture dishes and treated with 50 μM or 100 μM BT for 6 and 24 hrs. After treatment, the cells were washed with PBS (1×), collected by centrifugation after trypsinization, re-suspended in fresh PBS and incubated with 5 μM 5,6-carboxy-2′,7′-dichlorodihydrofluorescein diacetate (carboxy-H_2_DCFDA, C400, Invitrogen, Eugene, Oregon, USA) for 30 min at 37°C. The cells were washed twice with DPBS, re-suspended in an equal volume of DPBS and fluorescence measured with flow cytometry. Data was acquired on a BD Accuri C6 flow cytometer and analyzed using Accuri C6 software (BD Immunocytometry-Systems, San Jose, CA). Twenty thousand cells were analyzed for each sample. Subsequent cell viability assay with ascorbic acid pretreatment were performed (see cell viability assay above).

### Western blot analysis

Western blotting was carried out to analyze expression of effector caspase 3 and caspase 7, using specific antibodies. Cellular pro-survival markers (pAkt, pNF-κB p65), pro-apoptotic signaling markers (pP38 MAPK) and important cell cycle regulatory proteins such as p27Kip1 and p21^Cip1^ were also analyzed by western blotting. Additionally, NF-kB regulated genes involved in cell survival, e.g., IkBα, xIAP, bcl-2, bcl-xl and were analyzed by western blotting.

Cells were seeded into 100 mm^2^ tissue culture dishes (5 × 10^5^ cells/dish) and treated with 50 μM or 100 μM BT. Following 24 hrs of treatment, cells were harvested by trypsinization, washed with PBS and suspended in cell extraction buffer (Invitrogen, Carlsbad, CA) containing 10 mM Tris, pH 7.4, 100 mM NaCl , 1 mM EDTA, 1 mM EGTA, 1 mM NaF, 20 mM Na4P2O7, 2 mM Na3VO4, 1% Triton X-100, 10% glycerol, 0.1% SDS, 0.5% deoxycholate protease inhibitor cocktail and PMSF. Following heat denaturation, Lammli sample buffer along with β-mercaptoethanol was added to lysates and subjected to SDS-PAGE electrophoresis and immunoblotting. Following incubation with respective primary antibodies for overnight at 4°C, and appropriate secondary antibodies (Licor), the proteins on the blots were detected by Licor image analyzer.

### Autotaxin (ATX) assay

The phosphodiesterase activity of ATX was measured using a modification of the method of Razzell and Khorana [16]. ATX is secreted into media. After treatment with BT, cell-free supernatants were collected for ATX estimation. The cells were gently scraped off for analysis of cellular protein levels, according to the method of Lowry et al., [17]. The concentration of ATX was normalized with respect to the cell mass of samples in each well. To estimate ATX, cell free culture media (100 μL) was incubated with 100 μL substrate containing *p*-nitrophenylphosphonate (pNppp) at a final concentration of 5 mM prepared in 50 mM Tris–HCl buffer, pH 9.0. After 30 min incubation at 37°C, the reaction was stopped by the addition of 100 μL of 0.1 M NaOH solution. The reaction product was measured by reading the absorbance at 410 nm. The percent of ATX inhibition of treated cells was calculated against untreated cells.

### Statistical analysis

All data were expressed as mean ± SD. Comparisons between untreated and each treated group were performed by Student’s t–test. The significance level was set at p < 0.05.

## Results

### Cytotoxic effects of BT on ovarian cancer cell lines

**Figure 1:**
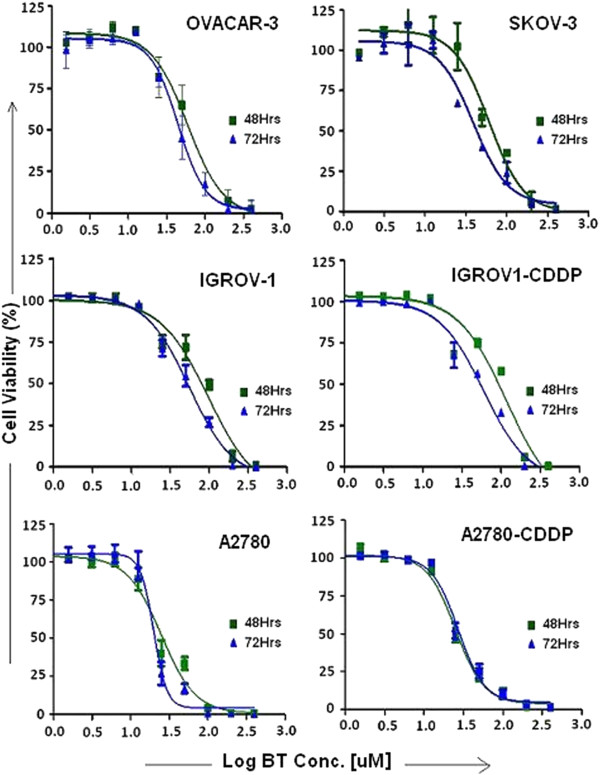
**Bithionol dose response curves.** Cytotoxic effects of BT on a panel of ovarian cancer cell lines with varying cisplatin sensitivities. Cells were treated with different concentrations of BT for 48 or 72 hrs. Cell viability was determined by PrestoBlue Cell Viability Reagent as described in Materials and Methods. Control cells (vehicle treated) were considered as 100% viable against which treated cells were compared. Experiments were performed in triplicate. Data was expressed as mean ± SD of triplicate experiments. Dose response curves to calculate IC_50_ values were plotted using Graph Pad Prism Software.

As shown in Figure 1, treatment with increasing concentrations of BT resulted in dose dependent reduction in cell viability in all the cell lines tested. At 72 hrs post treatment, the sensitivities to BT can be ranked from high to low as A2780 (IC_50_ - 19 μM) > A2780-CDDP (IC_50_ - 24 μM) > SKOV-3 (IC_50_ - 36 μM) > OVACAR-3 (IC_50_ - 44 μM) > IGROV-1(IC_50_ -55 μM) > IGROV1-CDDP (59 μM) (Table 1). Interestingly, cisplatin-resistant variants of A2780 and IGROV-1 showed near similar BT IC_50_ values to their cisplatin-sensitive variants, although significant difference were observed with cisplatin IC_50_ values (Table 2).

**Table 1:** **IC**_**50 **_**values for Bithionol in various ovarian cancer cell lines**

		**48 hrs**	**72 hrs**
1	OVACAR-3	59 ± 9	44 ± 7
2	SKOV-3	60 ± 2	36 ± 7
3	IGROV-1	98 ± 14	55 ± 12
4	IGROV-1 CDDP	117 ± 21	59 ± 8
5	A2780	26 ± 4	19 ± 3
6	A2780-CDDP	27 ± 3	24 ± 3

IC_50_ values (μM) for Bithionol - mean ± SD.

**Table 2:** **IC**_**50 **_**values for Cisplatin in isogenic ovarian cancer cell line pairs**

		**48 hrs**	**72 hrs**
1	IGROV-1	13 ± 1	3 ± 0.05
2	IGROV-1 CDDP	60 ± 8	18 ± 1
3	A2780	22 ± 3	10 ± 2
4	A2780-CDDP	42 ± 6	23 ± 4

IC_50_ values (μM) for Cisplatin - Mean ± SD.

IC_50_ values for Cisplatin in isogenic ovarian cancer cell line pairs consisting of Cisplatin-sensitive parental lines and stable Cisplatin-resistant sub-lines derived by *in vitro* selection with Cisplatin.

### Assessment of type of cell death induced by bithionol

#### Effect of BT on lactate dehydrogenase (LDH) activity (necrosis assessment)

**Figure 2:**
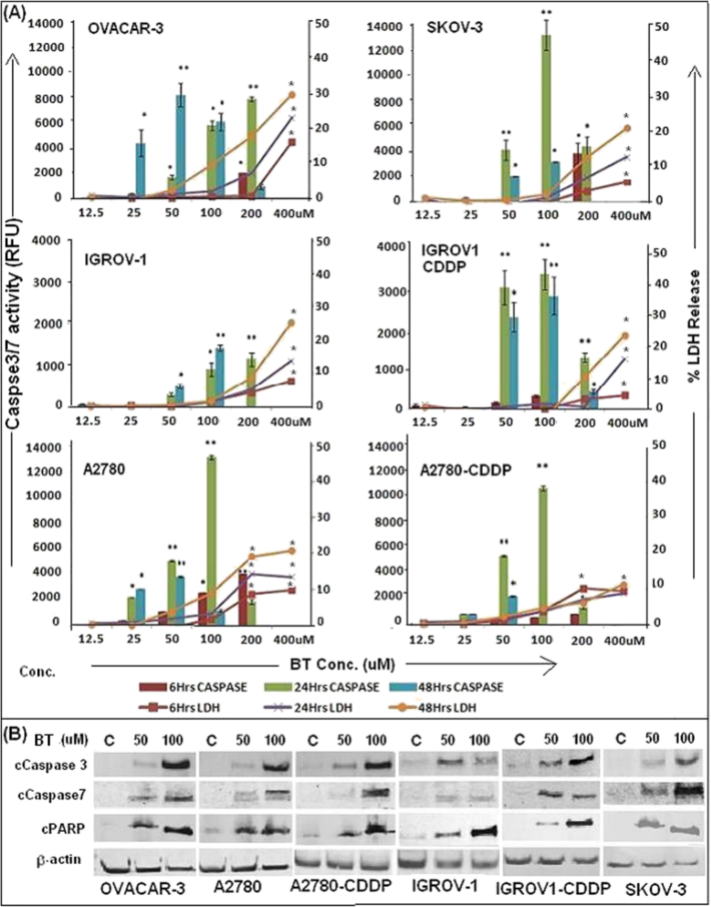
**Assessment of type of cell death induced by BT on various ovarian cancer cell lines. (A)** Effect of BT on casapses3/7 (columns) and LDH activities (line graph). Caspase 3/7 activity was measured using Caspase-Glo 3/7 Assay kit from Promega and LDH release was measured by using Cyto-Tox-One Homogenous Membrane Integrity kit (Promega). Cells were treated with BT for 6, 24 and 48 hrs. Vehicle treated (media with 1% DMSO) were considered as control against which treated cells were compared. Experiments were performed in triplicate. Data was expressed as mean ± SD of triplicate experiments. *p < 0.05 and **p < 0.01, as compared to control, Students” test. **(B)** Activation of Caspases 3 and 7 and degradation of PARP as shown by western blot analysis. Ovarian cancer cell lines were treated with BT at 50 μM or 100 μM for 24 hrs. Analysis of the expression of proteins in the lysates of treated and untreated cells (control) was carried out by PAGE and western blot analysis as described (Materials and Methods). Primary antibodies against activated caspase-3, caspase-7 and cleaved PARP were used. As an internal standard for equal loading, blots were probed with an anti -*β*- actin antibody.

Our results demonstrate that LDH release is dependent on BT concentration and treatment time. As shown in Figure 2A (line graph), at 6 and 24 hrs post treatment, no significant LDH release was observed at lower concentrations (12.5 μM - 100 μM), but only occurred at higher concentration (200 & 400 μM). However, at 48 hrs post-treatment, LDH release was observed even at lower concentration (100 μM BT) especially in OVACAR-3 and A2780 cell lines. All cell lines tested exhibited a similar trend.

### Effect of BT on caspase 3/7 activity (apoptosis assessment)

Our results demonstrate that BT induces caspase activity in all cell lines tested. Caspase activity was found to be dependent on time and concentration of BT. As shown in Figure 2A (column graph), at 6 hrs post treatment, caspase activity was observed only at 200 μM in all cell lines except A2780 which showed significant activity even at 50 μM BT. However, at 24 hrs post treatment, significant caspases activity was observed at lower concentrations (50 μM - 100 μM BT). At 48 hrs post treatment, caspase activity was still observed at lower concentrations but absent at higher concentrations. No caspase activity was observed at 400 μM BT at any time points.

Western blot analysis demonstrated significant expression of caspase 3 in all cell lines tested. Similarly, activation of caspase-7, as indicated by the appearance of a 20 kDa band, was observed in all BT treated cell lines. As compared to all cell lines, IGROV-1CDDP exhibited weak caspase-7 expression (Figure 2B). Caspases expression peaked at 24 hrs post treatment. The activation of proteolytic caspases following drug exposure resulted in the cleavage of 118 kDa PARP-1 protein into an 89 kDa fragment in all BT treated cell lines (Figure 2B). Untreated cells did not show any PARP cleavage. All cell lines exhibited similar results.

### Morphological hallmarks of apoptosis

**Figure 3:**
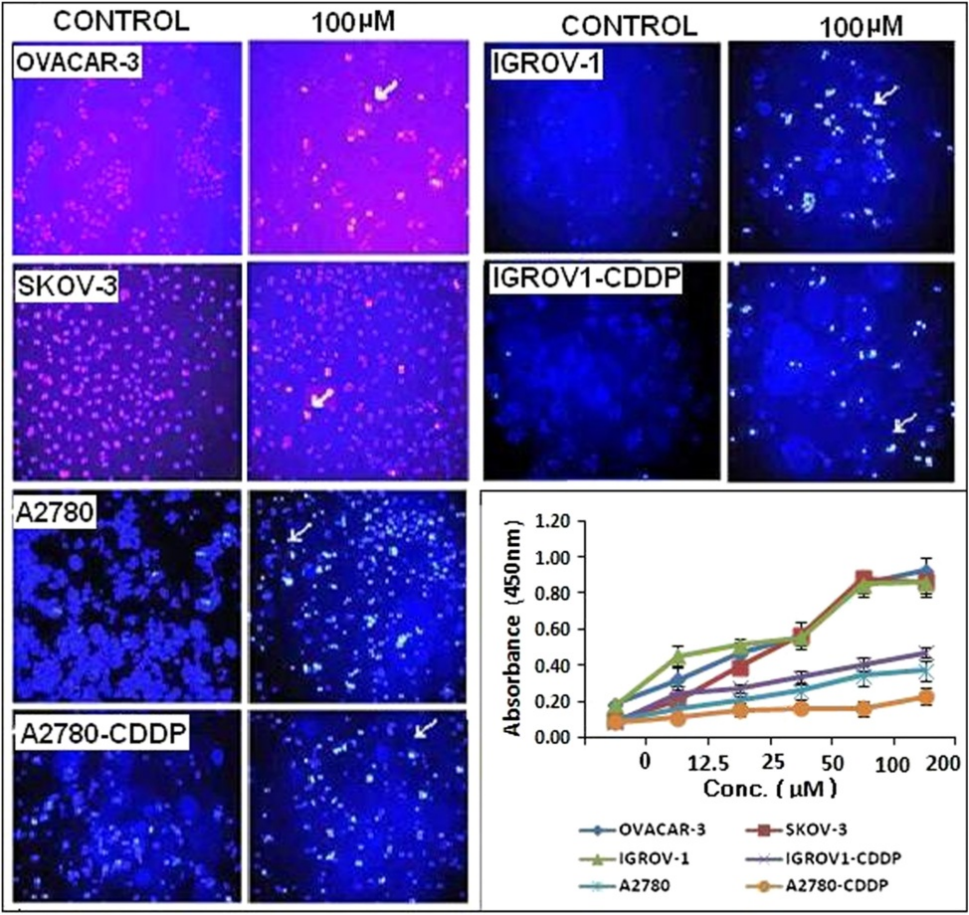
**Hoechst staining of cell to detect BT induced apoptosis.** Ovarian cancer cell lines were treated with 100 μM BT for 24 hrs. Treated/untreated cells were stained with Hoechst 33258 and visualized by fluorescence microscopy. Representative images were taken with an inverted microscope (Olympus H4-100, CCD camera) and 20× objective. Graph: Quantification of percent of apoptosis in terms of DNA fragmentation using Trevigen’s TACS® 2 TdT *in Situ* Apoptosis Detection Kit (TUNEL assay). Cells were treated with BT as explained earlier. At the end of the treatment time, labelled nucleotides were added and detected with HRP – HRP substrate (TACS-Sapphire) system. The absorbance was measured at 450 nm using a microplate reader, Multiskan (Thermo Scientifics). Experiments were performed in duplicate. Data was expressed as mean ± SD of duplicate experiments.

As shown in Figure 3, normal control cells stained very faintly with the Hoechst stain but treated cells had a stronger blue fluorescence indicative of apoptosis. Strong blue fluorescence indicates highly condensed chromatin, characteristic of apoptotic cells. These results are also confirmed by TUNEL assay which detects DNA fragmentation. As shown in Figure 3 (line graph), increased DNA fragmentation was observed with increasing BT concentrations in all the cell lines tested.

### Analysis of mitochondrial transmembrane potential

**Figure 4:**
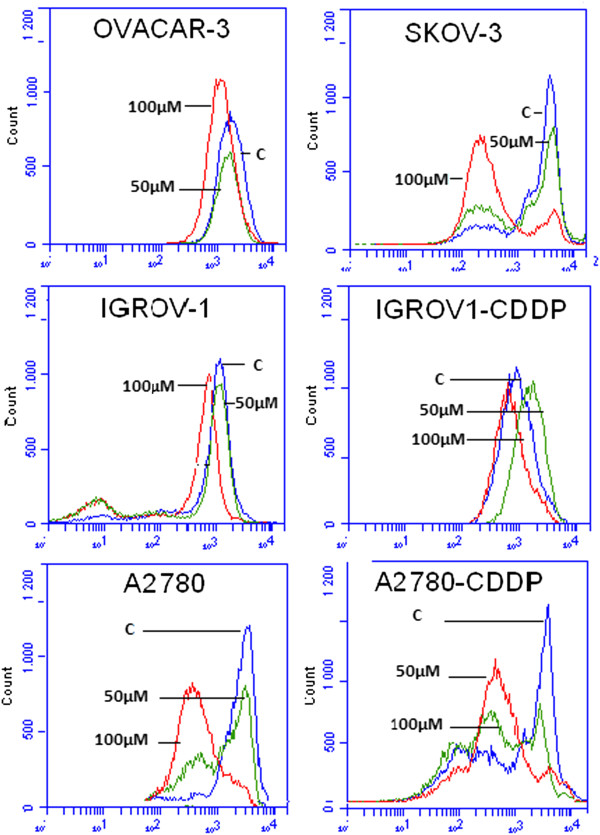
**Detection of loss of mitochondrial trans-membrane depolarization potential upon BT treatment.** Cell lines were treated with 100 μM BT for 24 hrs. Mitochondrial potential was determined by flow cytometry using Rhodamne-123. Data was acquired by BD’s Accuri C6 flow cytometer system and analyzed. Data was presented as relative-fluorescence intensity in a 2-dimensional FACS profile (standardized gating, 20,000 events). Loss of transmembrane potential was shown by shift in peaks in treated cells as compared to control (C– vehicle treated). All experiments were performed in triplicate.

BT treatment resulted in slight decrease in mitochondrial potential as early as 6 hrs post treatment. At 24 hrs post-treatment, significant mitochondrial loss was observed in all cell lines as indicated by shifts in peaks between untreated, 50 μM BT and 100 μM BT treated cells (Figure 4). As compared to OVACAR-3 and IGROV-1 and IGROV1-CDDP, loss of mitochondrial potential was greater in SKOV-3, A2780 and A2780-CDDP at 24 hrs post treatment.

### Mechanism(s) of BT induced cytotoxicity

#### Effect of BT on cell cycle in ovarian cancer cell lines

**Figure 5:**
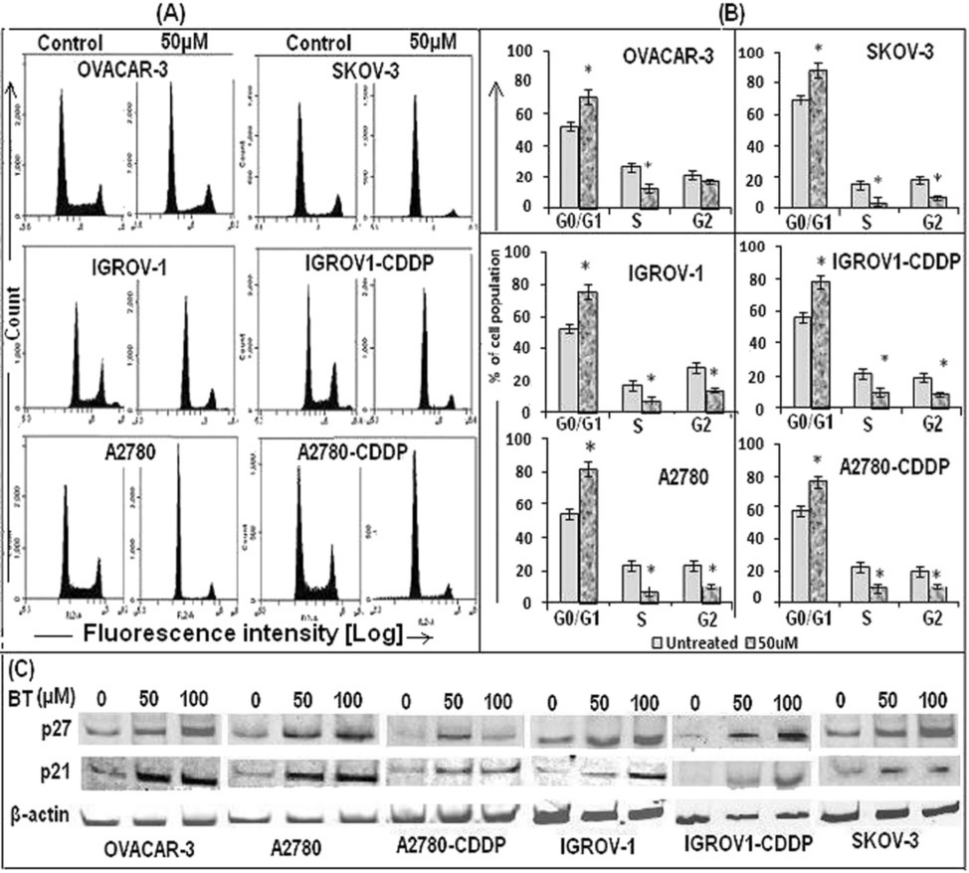
**Effect of BT on cell cycle progression of ovarian cancer cell lines. (A)** Cell cycle analysis was carried out by flow cytometry. Cells were treated with 50 μM BT for 24 hrs. At the end of the incubation period, cells were collected, fixed and stained with 50 μg/mL of propidium iodide and 100 μg/mL of RNase for 30 min at 37°C in the dark. Data was acquired on a BD Accuri C6 flow cytometer and analyzed. Twenty thousand events were analyzed for each sample. Appropriate gating was used to select standardized cell population. **(B)** Graphical representation of cell cycles analysis by FACS. Data was expressed as mean ± SD of triplicate experiments. *p < 0.05, as compared to untreated control, students’ t test. **(C)** Expression of cyclin-dependent kinase inhibitors such as p27^Kip1^ and p21^Cip1^ in BT treated cell lines, as analyzed by western blotting of cellular lysates using appropriate primary and secondary antibodies. Ovarian cancer cell lines were treated with BT at 50 μM or 100 μM for 24 hrs.

At 24 hrs post treatment, cell-cycle analysis of BT treated ovarian cancer cell lines revealed a significant increase in the G1-phase cell population with a concomitant decrease in S and G2 phases as compared to untreated control (Figure 5A and B). OVACAR-3 did not show significant change in G2 phase (p > 0.05).

Western blot analysis of cell cycle regulatory proteins revealed up-regulation of both P27 *(kip1)* and p21 upon BT treatment (Figure 5C).

### Effect of BT on ROS generation

**Figure 6:**
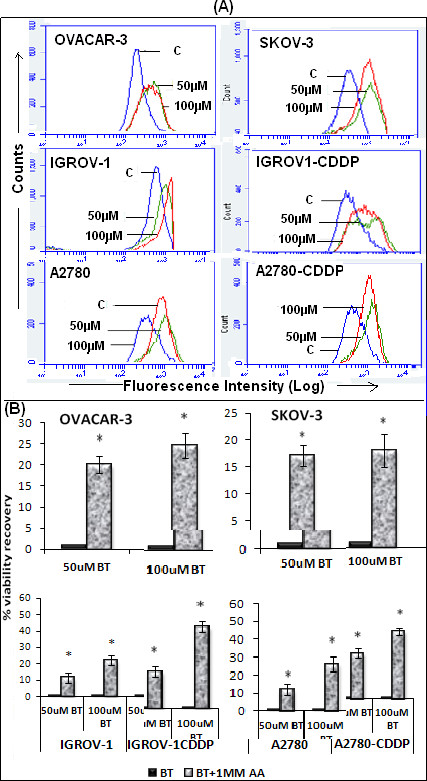
**Detection of intracellular ROS following BT treatment in ovarian cancer cell lines. (A)** Detection of ROS by flow cytometry. Cells (1 × 10^6^) were treated with 50 μM or 100 μM **BT** for 24 hrs. After treatment, cells were collected, washed and incubated with 5 μM C400 and analyzed by flow cytometry. Data was presented as relative-fluorescence intensity in a 2-dimensional FACS profile (standardized gating, 20,000 events). Enhanced ROS generation was shown by shift in peaks in treated cells as compared to control (labeled as ‘**C**’). All experiments were performed in triplicate. **(B)** Effect of antioxidant ascorbic acid on BT treated cells. Cells were pre-treated with 1 mM ascorbic acid for 2 hrs before addition of drug and further incubated for 48 hrs with both BT and ascorbic acid. Cell viability was determined by PrestoBlue reagent. Control (untreated) cells were considered as 100% viable against which treated cells were compared. The results represent % viability recovery when compared with 100 μM BT treated cells. Experiments were performed in triplicate. Data was expressed as mean ± SD of triplicate experiments. *p < 0.05 and **p < 0.01, as compared to control, Students’ t test.

Cells treated with BT showed ROS generation as early as 6 hrs post treatment. This was more remarkable when treatment was extended up to 24 hrs. As shown in Figure 6A, elevated ROS levels were observed in all cell lines as indicated by shift in peaks between untreated, 50 μM BT and 100 μM BT treated cells.

Follow up cell viability assays in the presence of antioxidant ascorbic acid, demonstrated at least a 20-30% restoration of cell viability in the presence of 1 mM ascorbic acid in OVACAR-3, SKOV-3, IGROV-1 and A2780 cells. Interestingly, greater restoration of cell viability was observed in cisplatin-resistant variants of IGROV-1 and A2780. In IGROV-1CDDP, 47% cell viability was restored and A2780-CDDP showed 40% restoration (Figure 6B).

### Effect of BT on pro-survival (pAkt, NF-κB) and pro-apoptotic (pP38) signalling molecules

**Figure 7:**
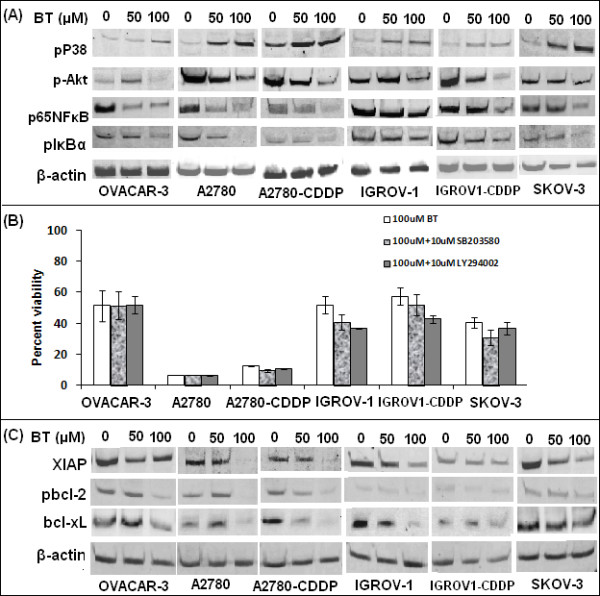
**Effect of BT on pro-apoptotic (pP38) and pro-survival (pAkt, NF-κB) signalling molecules. (A)** Cells were treated with 50 μM or 100 μM BT for 24 hrs, proteins extracted, subjected to electrophoresis and detected by western blotting using primary antibodies specifically recognizing the phosphorylated active forms of these proteins. As an internal standard for equal loading, blots were probed with an anti *β*-actin antibody. **(B)** Effect of p38 inhibitor SB203580 and pAkt inhibitor LY294002 on BT treated cells. To assess role of p38 and pAkt activation in BT induced cytotoxicity, cells were treated with 100 μM BT in presence of 10 μM of SB203580 or 10 μM LY294002 (non-toxic conc.’s) for 48 hrs and cell viability determined by presto blue cell viability reagent. The results represent % viability when compared with untreated cells. Data was expressed as mean ± SD of triplicate experiments. **C**. Effect of BT treatment on NF-kB regulated cell survival genes such as pIkBα, xIAP, pbcl-2, bcl-xL, as analyzed by western blotting of cellular lysates.

As shown in Figure 7A, western blot analysis revealed significant activation of pro-apoptotic marker, p38, when cells were treated with BT for 24 hrs. However, a cell viability assay using SB203580 pre-treatment (an inhibitor of p38) did not restore cell viability (Figure 7B).

Western blot analysis of pro-survival marker pAkt showed decreased expression at 24 hrs post-BT treatment in all cell lines except for OVACAR-3 and IGROV-1 where increased expression was observed at 50 μM but decreased at 100 μM BT (Figure 7A). Additionally, a cell viability assay using LY294002 pre-treatment (an inhibitor of pAkt) neither enhanced BT cytotoxicity nor restored cell viability at 48 hrs post BT treatment.

Pro-survival marker, phospho-NF-κB p65, showed decreased expression at 24 hrs post-BT treatment in all cell lines at 100 μM BT (Figure 7A). Interestingly, down-regulation of several genes regulated by NF-κB (pIkBα, XIAP, pbcl-2, bcl-xL) was observed in all cell lines (Figure 7C). Expression of pro-survival marker XIAP, a direct inhibitor of executioner caspases, such as caspase-3, was down-regulated within 24 hrs following the BT treatment in all the cell lines (Figure 7C).

Activation of NF-κB occurs via phosphorylation of IκBα at Ser32 and Ser36. This is followed by proteasome-mediated degradation resulting in release and nuclear translocation of active NF-κB, where it regulates expression of several pro-survival or pro-apoptotic proteins, e.g., pIkBα, pbcl-2, bcl-xL, xIAP. Expression of pNFkB, pIkBα, XIAP, pbcl-2 and bcl-xL were assessed by western blotting. pNFkB was detected using a specific antibody that detects NF-κB p65 only when phosphorylated at Ser536. Similarly, expression of phosphoIkBα was detected using a monoclonal antibody that detects endogenous levels of IκBα only when phosphorylated at Ser32. As described in Figure 7A, pro-survival marker, phospho-NF-κB p65, showed decreased expression at 24 hrs post BT treatment in all cell lines at 100 μM BT. Similarly, pIκBα levels were reduced at 24 hrs post-treatment. The extent of decrease varied between cell lines with a significant decrease observed in A2780, SKOV-3 and OVACAR-3. Compared to all cell lines, A2780-CDDP showed weak expression of pIkBα at all concentrations. Interestingly, down-regulation of several genes regulated by NF-κB (pIkBα, XIAP, pbcl-2, bcl-xL) was observed in all cell lines (Figure 7C). BT at 100 μM consistently inhibited pbcl-2 and bcl-xL in all cell lines. Phospho-Bcl-2 was detected using an antibody that detects Bcl-2 only when phosphorylated at threonine56. Expression of pro-survival marker XIAP, a direct inhibitor of executioner caspases, such as caspase-3, was down-regulated within 24 hrs following the BT treatment (100 μM) in all the cell lines (Figure 7C).

### Effect of BT on autotaxin inhibition

**Figure 8:**
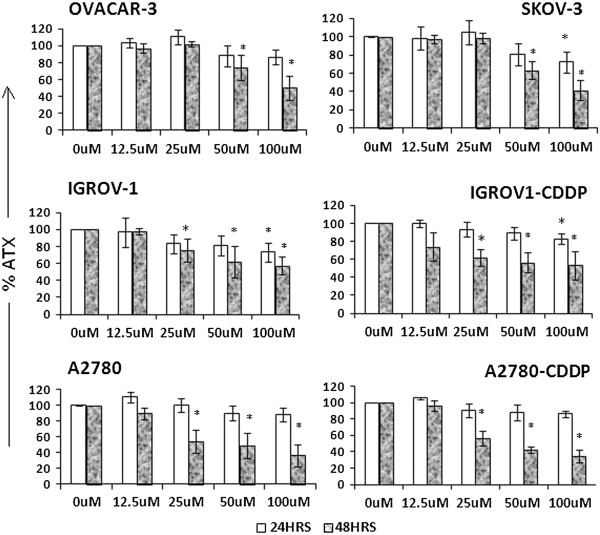
**Effect of BT on ATX secretion in ovarian cancer cell lines.** Cells were treated with BT at different concentrations for 24 and 48 hrs. At the end of the treatment, ATX was measured from culture media by a colorimetric assay using *p*-nitrophenylphosphonate (pNppp) as substrate. The ATX concentration is normalized against cell number. The percent of ATX inhibition of treated cells was calculated against untreated cells. Experiments were performed in triplicate. Data was expressed as mean ± SD of triplicate experiments. *p < 0.05 as compared to control; **p < 0.01 as compared to control.

BT treatment significantly inhibited ATX in all the cell lines tested (Figure 8). BT induced ATX inhibition was time dependent as more inhibition was observed at 48 hrs post treatment than at 24 hrs. Approximately 40-60% inhibition was observed at 100 μM BT at 48 hrs post treatment in all cell lines tested. The extent of ATX inhibition was nearly similar in all cell lines.

## Discussion

Drug resistance is a major cause for ovarian cancer recurrence. New drug discovery requires significant resources and time. Alternatively, the concept of ‘drug repurposing’ appears promising. In the present study, we explored the antitumor potential of BT in pre-clinical ovarian cancer model. BT was tested against a panel of ovarian cancer lines exhibiting varying sensitivities to cisplatin. Our results demonstrate the cytotoxic effects of BT towards all the ovarian cancer cells lines tested with IC_50_ values ranging from 19 μM to 60 μM, at 72 hrs post treatment. Interestingly, BT IC_50_ values were almost indistinguishable between cisplatin-sensitive and cisplatin-resistant variants of isogenic ovarian cancer cell line pairs, although cisplatin IC_50_ values varied significantly. These results are significant when considering that clinically, all recurrent ovarian cancers will eventually be platinum-resistant. Interestingly, BT IC_50_ values observed for various ovarian cancer cell lines are significantly below the clinically tolerable doses of BT for humans. In several published studies, chronic BT dosing up to 50 mg/kg every other day was well tolerated with the 40 mg/kg dose level best tolerated. Fifty mg/kg in three divided alternate daily doses for 5 days will maintain serum levels of BT in the range of 140 to 550 μM in rabbits, dogs and humans [13,18]. Based on the fact that BT exerts similar cytotoxic effects on cisplatin-sensitive and resistant ovarian cancer cell lines with clinically tolerable IC_50_ values, it is reasonable to speculate that BT may be useful in halting ovarian cancer cell growth irrespective of the sensitivity that cells may display to cisplatin, and this merits further exploration.

It is well known that invalid apoptosis pathway has often been one of the hallmarks of cancer cells and an important cause of resistance to cytotoxic agents [19]. It is therefore essential to focus on type of cell death induced by therapeutic agents. Ability to induce apoptosis is a critical factor for effective treatment against cancer [20]. Previous reports show the inhibitory effect of BT on cervical cancer cell growth via induction of caspase 3/7 activity [14]. Our results also indicate that ovarian cancer cells undergo apoptosis upon BT treatment initially at lower concentrations. Hallmarks of apoptosis, such as nuclear condensation, DNA fragmentation, and loss of mitochondrial potential, were observed further demonstrating that BT triggers apoptosis in ovarian cancer cells. However, at higher concentrations, no caspase activity was detected while LDH was detected, indicating that cells die via necrosis at higher concentrations. The ability of BT to induce cell death via apoptosis makes this drug a good candidate for the treatment of ovarian cancer.

This study also demonstrates that BT induces apoptosis in ovarian cancer cells via activation of proteolytic effector caspases such as Caspase 3 and 7 and subsequent cleavage/inactivation of PARP-1 (involved in DNA repair). Apoptosis is known to be mediated by two pathways, the extrinsic (death receptor) and the intrinsic (mitochondrial). The majority of anticancer (cytotoxic) drugs induce apoptosis via the intrinsic (mitochondrial - cytochrome c/Apaf-1/caspase-9 pathway) [21,22]. Mitochondria are considered to be both a source and a target of ROS. Although we did not focus on which apoptotic pathway was induced by BT, decreased mitochondrial transmembrane potential following BT treatment implicates the intrinsic (mitochondrial) pathway. Disruption of mitochondrial potential can lead to oxidation of mitochondrial pores by ROS, resulting in release of cytochrome C into the cytosol [23]. Cytochrome C, Apaf1 (apoptotic protease activating factor-1) and dATP then form an apoptosome to which procaspase-9 is recruited and activated. Caspase-9 in turn activates downstream effector caspases −3 and −7 which execute the final steps of apoptosis.

We observed a switch from apoptosis to necrosis with increasing BT concentrations. Apoptosis is a carefully regulated, energy-dependent process that involves a complex cascade of events resulting in cell death. It is dependent on availability of ATP, which in turn depends on the correct function of mitochondria. As mentioned in our manuscript, BT causes mitochondrial transmembrane depolarization, thus affecting mitochondrial function. This disruption may cause ATP depletion to a level that is insufficient for cell survival, thus switching from apoptosis to necrosis. Additionally, reactive oxygen species (ROS) are known to cause apoptosis or necrosis, depending on the amount and type of ROS generated [24]. We postulate that high concentrations of BT lead to increased ROS, ultimately causing severe cellular injury. High levels of ROS can inhibit apoptosis by inactivating caspases by oxidation of their thiol groups. Furthermore, ROS can affect mitochondrial energy (ATP) production causing depletion of ATP. These events would ultimately switch cells to necrosis.

Inhibition of the cell cycle is a known target for the treatment of cancer [25-28]. Anticancer agent may cause cell cycle arrest via altering the regulation of cell cycle machinery. Various regulatory proteins, including cyclin E, cyclin D1, cyclin D2, cyclin A, CDK2, CDK4 and the CDK inhibitors p27^Kip1^ and p21^Cip1^ are known to regulate cell cycle. It is well known that kinase activities of CDK-cyclin complexes are essential for progression of cell cycle at many check points [29-31]. p21^Cip^ is regarded as universal inhibitor of cyclin-CDK complexes [29-31], thus blocking the entry of cells at the G_1_-S-phase transition checkpoint and induce apoptosis [32]. Our data demonstrate that BT treatment resulted in G1-phase cycle arrest and up-regulation of the expression of p27^Kip1^ and p21^Cip1.^ Increased expression of CDK inhibitors p21^cip1^ and p27^kip1^ may result in increased association with CDKs, thus inhibiting their activity. The cascade of downstream events in response to BT treatment may lead to blockage of the cell cycle at the G1-to-S phase transition, and thus halting ovarian cancer cell growth. Additionally, cell cycle arrest following BT treatment could be ROS mediated. We showed that BT enhanced ROS generation. ROS mediated inactivation of CDKs by via oxidation [33] and enhanced expression of p21 can cause cell cycle arrest in G1- and S-phases resulting in reduced cellular proliferation. ROS mediated DNA damage is known to cause stabilization and elevation of known tumor suppressor protein, p53, which in turn induces and enhances the synthesis of p21 [33]. As mentioned earlier, p21 is known inhibitor of CDK activity. These observations suggest that cell cycle regulation is one of the mechanisms of action of BT in ovarian cancer cells.

Increased ROS generation can be frequently observed in cells subjected to anticancer drugs such as paclitaxel, cisplatin, doxorubicin [34,35]. Accumulation of ROS inside the cell may result in apoptosis or terminal differentiation [36]. Our results demonstrate significant generation of ROS in BT treated cells as compared to untreated cells in both a concentration and time dependent fashion. In order to ascertain role of ROS in BT induced cytotoxicity, we performed a cell viability assay in the presence of BT and antioxidant, ascorbic acid. Our results demonstrate a significant restoration of cell viability in the presence of 1 mM ascorbic acid in all cell lines tested. Interestingly, cisplatin-resistant variants of IGROV-1 and A2780 demonstrated greater responses to ascorbic acid pre-treatment than their cisplatin-sensitive counterparts. These observations imply a significant role of ROS in BT mediated cytotoxicity, and more so in cisplatin-resistant cell lines. This unique effect of BT on ROS generation in cisplatin-resistant cells implies that BT could have a role in the treatment of platinum-resistant ovarian cancer, either alone or in combination with other cytotoxic drugs.

Reactive oxygen species are known to modify signalling molecules important in cellular survival such as Akt1, and transcription factors including NF-kB, due to the presence of redox-sensitive cysteine or methionine groups that are susceptible to oxidation [37]. It is widely reported that cisplatin-resistant cell lines maintain high levels of Akt and NF-kB as compared to cisplatin-sensitive cell lines [38]. Keeping in mind the greater role of ROS generation observed in cisplatin resistant variants upon BT treatment, it may be possible that modification of pro-survival molecules such as Akt and NF-kB via oxidation may be a possible mechanism of action of BT, especially in cisplatin-resistant cell lines.

To further define key signalling responses of ovarian cancer cells to treatment with BT, we analyzed the expression and activation/phosphorylation of cellular markers involved in pro-apoptotic (p38) or pro-survival (pAkt, NF-kB) signalling. Immunoblotting of PAGE-separated cellular lysates revealed sustained activation of pP38 MAPK upon BT treatment. In order to assess the role of pP38 signalling in BT induced cytotoxicity, a cell viability assay was performed in the presence of a p38 inhibitor, SB203580. Pre-treatment with the p38 inhibitor did not restore cell viability when cells were treated with BT. These results rule out any significant role for p38 MAPK signalling in BT mediated cytotoxicity.

Activation of the PI-3 K/Akt pathway has been shown to induce resistance to apoptosis induced by a number of drugs and has been linked to cisplatin resistance in ovarian cancer cell lines [39,40]. In view of this, we studied the expression of pAkt upon BT treatment. Significant down-regulation of pAkt expression was observed at 24 hrs post BT treatment. It has been reported that Akt inactivation is essential for drug sensitivity [41,42]. In order to understand whether further inactivation of Akt can enhance the effectiveness of BT, we performed cell viability assays in the presence of PI3k inhibitor LY294002. LY294002 neither enhanced BT cytotoxicity nor restored the cell viability at 48 hrs post BT treatment. These results show that the Akt pathway may not mediate BT cytotoxicity in ovarian cancer cell lines.

Inhibition of the IKK/NF-*κ*B activation pathway is considered an effective target for many anticancer drugs [43]. NF-kB inhibition in cancer cells has been shown to enhance chemotherapeutic response [44,45]. BT has also been reported to inhibit NF-kB signalling via inhibition of IkBα phosphorylation *in vitro*[14]. Given the relevance of the NF-κB pathway in cancer, we assessed the effect of BT on phospho-NF-κB p65 and subsequent effect on NF-kB regulated proteins such as pIkBα, pbcl-2, bcl-xL, xIAP. Immunoblot analyses of whole cell lysate reveal decreased phospho-NF-κB p65 expression with increasing treatment time. BT treatment also down-regulated the expression of pIkBα. Suppression of proliferation, induction of apoptosis and G1/S cell cycle arrest can all be due to inhibition of phosphorylation of NF-kB and IkBα. BT can affect the DNA binding activity of NF-kB directly via oxidation by ROS [46] and/or indirectly by inhibiting phosphorylation of NF-κB [47] and IkBα. Phosphorylation of p65 at ser536 is essential for the DNA binding activity of NF-κB and it is known to be mediated via the PI3-kinase pathway [48]. Because BT also decreased pAkt expression, BT appears to indirectly reduce the DNA binding activity of NF-κB and affect the expression of NF-κB regulated anti-apoptotic proteins such as pIkBα, pbcl-2, bcl-xL, xIAP. Indeed, we observed that NF-kB regulated proteins XIAP, bcl-xl, pbcl2 were down regulated upon BT treatment. XIAP is known to prevent apoptosis through up-regulation of PI3k/Akt cell survival signalling pathway [49]. Down–regulation of XIAP induces apoptosis and increases cisplatin sensitivity [49]. Inhibition of Bcl-xl may increase sensitivity to drugs such as carboplatin [50]. Expression of Bcl-2 is important in protection from drug-induced apoptosis in ovarian cancer thereby contributing to chemo-resistance [51,52]. These reports implicate NF-kB as a desirable target for anticancer agents in ovarian cancer. Our results demonstrate inhibitory effect of BT on NF-kB regulated proteins in ovarian cancer cell lines. BT treatment may promote apoptotic role for NF-κB by repressing anti-apoptotic gene expression. Our results indicate an important role for NF-kB in BT induced cytotoxicity. However, further studies are required to confirm role of NF-kB in the anti-tumor effects of BT in ovarian cancer cell lines.

Autotaxin (ATX) inhibition was considered major mechanism of action of BT. Previously BT was shown to inhibit solid tumor growth in several preclinical cancer models by targeting ATX [12,13]. ATX plays a major role in modulation of the cellular process through its enzymatic production of lysophosphatidic acid (LPA). ATX is known to increases the aggressiveness and invasiveness of transformed cells, and directly correlates with tumor stage and grade in several human malignancies, including ovarian cancer [53,54]. ATX was shown to delay carboplatin induced apoptosis in ovarian cancer cells [55]. ATX inhibition was a proposed mechanism of action of BT in a melanoma model via inhibition of cell migration and invasion [13]. Given the significance of ATX in ovarian cancer [55-58], we studied the effect of BT on ATX in a panel of ovarian cancer cell lines. Our results clearly demonstrate significant inhibition of ATX in a concentration and time dependent fashion. ATX/LPA stimulate the PI3-K, Akt, and ERK pathways and cause the activation of Rho and Rac [59]. These pathways facilitate cell division, survival, and migration [60,61]. BT may inhibit cell survival directly via inhibition of ATX or indirectly via inhibition of PI3-K, Akt or NF-kB pathways. Additionally, ATX is known to act as antioxidant, thus, protecting cells from oxidative stress [62]. The fact that BT treatment reduced ATX activity would imply that treated cells are exposed to a higher oxidative stress, eventually leading to apoptosis or necrosis. In view of the significance of ATX in chemoresistance in a majority of widely used chemotherapeutic agents, ATX inhibition or the LPA pathway can be considered as a significant therapeutic target. In our studies, we also observed a significant inhibition of ATX by BT.

Based on our findings, BT affects cells by causing mitochondrial dysfunction, ROS generation, cell cycle arrest and ATX inhibition, ultimately leading to cell death (apoptosis at low concentrations and necrosis at higher concentrations). BT appears to be a viable therapeutic agent against ovarian cancer cell lines *in vitro*. Further exploration of its anti-tumor potential in ovarian cancer animal xenograft model is essential before proceeding to clinical trials. Additionally, it is interesting to focus on synergistic, additive or antagonistic effects of BT in combination with other standard chemo drugs. These studies are currently underway.

## Conclusions

We demonstrated the ability of BT to exert cytotoxic effects on a panel of ovarian cancer cell lines regardless of their cisplatin sensitivities. BT IC_50_ values observed in various ovarian cancer cell lines are well below the clinically tolerable doses of BT for humans. BT was shown to induce cell death via apoptosis. The mechanism(s) of actions appears to be via cell cycle regulation, ROS generation, NF-kB inhibition and ATX inhibition. ROS generation appears to be major mechanism of BT cytotoxicity in cisplatin-resistant variants. Agents causing cell cycle mediated apoptosis; NF-kB and ATX inhibition are already considered ideal candidates for the treatment of ovarian cancer. Because BT was shown to exhibit these desirable properties in in vitro, it is being further explored as an effective therapeutic agent in mice ovarian cancer xenograft model, either alone or in combination. In summary, the present study provides preclinical data supporting the possible therapeutic role of BT in the treatment of recurrent platinum-resistant ovarian cancers.

## Abbreviations

BT: Bithionol; ATX: Autotaxin; IC50: Half maximal inhibitory concentration; ROS: Reactive oxygen species; NF-κB: Nuclear factor kappa B; MAPK: Mitogen-activated protein kinase; XIAP: x linked inhibitor of apoptosis.

## Competing interests

The authors declare that they have no competing interests.

## Authors’ contributions

VA performed experimental procedures; LB and VA are responsible for study design, interpretation of results and writing of manuscript. Both authors read and approved the final manuscript.

## Pre-publication history

The pre-publication history for this paper can be accessed here:

http://www.biomedcentral.com/1471-2407/14/61/prepub
